# Altered local and matrix functional connectivity in depressed essential tremor patients

**DOI:** 10.1186/s12883-021-02100-3

**Published:** 2021-02-11

**Authors:** Xiyue Duan, Zhou Fang, Li Tao, Huiyue Chen, Xiaoyu Zhang, Yufen Li, Hansheng Wang, Aotian Li, Xueyan Zhang, Ya Pang, Min Gu, Jiahui Wu, Fajin Lv, Tianyou Luo, Oumei Cheng, Jin Luo, Zheng Xiao, Weidong Fang

**Affiliations:** 1 Department of Radiology, The First Affiliated Hospital of Chongqing Medical University, No. 1 Youyi Road, Yuzhong District, Chongqing, 400016 China; 2 Department of Neurology, The First Affiliated Hospital of Chongqing Medical University, Chongqing, 400016 China

**Keywords:** Essential tremor, Depression, Regional homogeneity, Functional connectivity, Resting-state functional magnetic resonance imaging

## Abstract

**Background:**

Depression in essential tremor (ET) has been constantly studied and reported, while the associated brain activity changes remain unclear. Recently, regional homogeneity (ReHo), a voxel-wise local functional connectivity (FC) analysis of resting-state functional magnetic resonance imaging, has provided a promising way to observe spontaneous brain activity.

**Methods:**

Local FC analyses were performed in forty-one depressed ET patients, 49 non-depressed ET patients and 43 healthy controls (HCs), and then matrix FC and clinical depression severity correlation analyses were further performed to reveal spontaneous neural activity changes in depressed ET patients.

**Results:**

Compared with the non-depressed ET patients, the depressed ET patients showed decreased ReHo in the bilateral cerebellum lobules IX, and increased ReHo in the bilateral anterior cingulate cortices and middle prefrontal cortices. Twenty-five significant changes of ReHo clusters were observed in the depressed ET patients compared with the HCs, and matrix FC analysis further revealed that inter-ROI FC differences were also observed in the frontal-cerebellar-anterior cingulate cortex pathway. Correlation analyses showed that clinical depression severity was positively correlated with the inter-ROI FC values between the anterior cingulate cortex and bilateral middle prefrontal cortices and was negatively correlated with the inter-ROI FC values of the anterior cingulate cortex and bilateral cerebellum lobules IX**.**

**Conclusion:**

Our findings revealed local and inter-ROI FC differences in frontal-cerebellar-anterior cingulate cortex circuits in depressed ET patients, and among these regions, the cerebellum lobules IX, middle prefrontal cortices and anterior cingulate cortices could function as pathogenic structures underlying depression in ET patients.

**Supplementary Information:**

The online version contains supplementary material available at 10.1186/s12883-021-02100-3.

## Background

Essential tremor (ET) has been gradually accepted as a heterogeneous disorder; in addition to the characteristic upper limb action tremor, ET has been linked to a variety of other motor and non-motor symptoms [1, 2] . In recent years, psychiatric and affective disturbances, particularly depression with ET, have been constantly studied and reported [3, 4]. However, it remains unclear whether depression in ET is associated with brain activity changes, such as depression in Parkinson’s disease (PD), or is merely a secondary mental phenomenon due to disabling tremor.

Recently, resting-state functional magnetic resonance imaging (RS-fMRI) has been suggested to be the most promising method to study brain activity changes in various neurologic and neuropsychiatric diseases, including neurodegenerative diseases, major depressive disorder [5] and ET. Our previous studies examining the local [6], seed-based [7] and network [8] functional connectivity (FC) of RS-fMRI have demonstrated that cerebellar-thalamic-cortical pathway dysfunction is linked to tremor and cognitive impairment in ET patients. Among these FC analytical methods, local FC analysis has the benefits of evaluation of spontaneous neural synchronization activity, easy interpretation of the results, and high test-retest reliability; it does not require any a priori hypotheses and has gained increased attention. However, to our knowledge, no studies have employed local FC analysis to investigate the underlying spontaneous brain activity changes in depressed ET patients.

In this study, regional homogeneity (ReHo) was adopted as a measure to explore local FC differences among depressed ET patients, non-depressed ET patients and healthy controls (HCs). Then, to further explore mutual relationships of significant changes in ReHo clusters, a matrix FC and clinical depression characteristics correlation analyses were performed. We expected to find alterations in ReHo in some brain areas involving depression and ET, particularly in the frontal-cerebellar-anterior cingulate cortex circuits.

## Materials and methods

### Subjects

Forty-one (26 male, 15 female) depressed ET patients, 49 non-depressed ET patients and 43 age- and sex-matched healthy controls (HCs) were selected from a study exploring the clinical and imaging biomarkers of ET (NSFCQ and NSFC: cstc2014jcyjA10047 and 81,671,663; January 2009 to present; a total of 331 ET patients have been enrolled). Each subject signed an informed consent form approved by the ethics committee of the First Affiliated Hospital of Chongqing Medical University (Chongqing, China), and the study was performed in accordance with the Declaration of Helsinki of the World Medical Association. All of the patients fulfilled the following criteria: 1) the patients met the diagnosis of definite or probable ET according to the Movement Disorders Consensus Criteria [9], and all of the patients had annual follow-ups through the outpatient department or by telephone; 2) the probable ET patients were followed up for at least 3 years to confirm the diagnosis; 3) the patients had an onset age between 18 and 55 years old, and patients with earlier or later onset were not included; 4) the patients were not treated with any anti-ET or anti-depressant medications before the baseline fMRI scan (only the baseline fMRI scan data were used in this study; the follow-up fMRI scan data were not included); 5) the patients were without any apparent cognitive impairment (Mini-Mental State Examination (MMSE) scores > 24) and were right-handed; 6) the patients presented with moderate or greater amplitude kinetic tremor (tremor rating ≥ 2) during at least three tests; 7) the patients were without PD, dystonia, psychogenic tremor, thyroid disease, stroke, epilepsy, head injury or any other neurological dysfunction; 8) the image quality met a root mean square head movement less than 0.5 mm and a frame-wise displacement of head movement < 50% of volumes (115 volumes); and 9) the depressed ET patients met the Diagnostic and Statistical Manual of Mental Disorders version four (DSM-IV) criteria [10]; that is, all of the patients had to have one or both of the two main symptoms (depressed mood, loss of interest or pleasure) that had lasted for more than two weeks.

The depression severity of each patient was evaluated by the 17-item Hamilton Depression Rating Scale (HDRS-17) [11], and all patients with scores of at least 7 points [12] were considered depressive. Tremor severity was assessed with the Fahn-Tolosa-Marin Tremor Rating Scale (TRS) [13] and the Essential Tremor Rating Assessment Scale (TETRAS) [14]. The Hamilton Anxiety Rating Scale (HARS-14) [15] assessed the anxiety severity of all of the participants. The MMSE was used to briefly assess cognitive function and to screen for dementia.

### MRI acquisition and preprocessing of resting-state fMRI data

Resting-state fMRI images, 3D T1-weighted images and T2-FLAIR images were acquired using a GE Signa Hdxt 3-T scanner (General Electric Medical Systems, Milwaukee, WI, USA); for detailed parameters, see text S1. Data preprocessing was conducted using DPABI: a toolbox for data processing & analysis for brain imaging, version 4.2 (http://rfmri.org/dpabi), as previously described [6], and detailed data-preprocessing steps are provided in text S2.

### Local FC analysis

DPABI was also adopted to compute the ReHo, as Zang et al. [16] and our previous studies [6] described. Briefly, individual ReHo maps were obtained by calculating Kendall’s coefficient of concordance (KCC) between each given voxel and those of its nearest neighboring voxels (26 voxels) in a voxel-wise manner. Then, to reduce the influence of individual ReHo variation and for standardization purposes, the individual ReHo maps were converted to *z*ReHo maps by subtracting the whole brain average ReHo value and dividing by the standard deviation of ReHo values across all of the brain voxels. Third, the *z*ReHo maps were smoothed with a Gaussian kernel of 4 × 4 × 4 mm^3^ full width at half maximum (FWHM). Finally, ANOVA and post hoc two-sample *t*-tests in a pair-wise manner within the areas identified by ANOVA were used to identify the ReHo changes among the three groups with Gaussian random field (GRF) multiple comparison corrections.

### Matrix FC analysis

To further explore the mutual relationship of significant changes, ReHo clusters, using the peak locations of these clusters as the centers, and spherical region of interests (ROIs) with a radius of 3 mm were defined. Then, the individual FC matrix was generated by calculating the Pearson’s correlation coefficient between the time courses of each ROI and the remaining ROIs. Third, Fisher’s *z*-transformation was performed to improve the normality of the Pearson’s correlation coefficient, and the *z*FC matrices were used for subsequent analyses. Finally, ANOVA and post hoc two-sample *t*-tests in a pair-wise manner within the areas identified by ANOVA were used to identify the inter-ROI FC changes among the three groups with Bonferroni’s multiple comparison correction.

### FC-depression correlation analysis

To identify whether brain region ReHo values or significant changes in inter-ROI FC values were related to the clinical depression profiles of the depressed ET patients, a voxel-wise Pearson’s correlation analysis between the individual ReHo maps and the HDRS-17 scores of the depressed ET patients was performed with GRF multiple comparison corrections, while an ROI-wise Pearson’s correlation analysis between the significant changes inter-ROI FC values and the HDRS-17 scores of the depressed ET patients was also performed with Bonferroni’s multiple comparison correction.

Furthermore, the demographic and clinical information was analyzed by descriptive statistics and presented as the means and deviations. Kolmogorov-Smirnov tests were performed to assess the normality of these data. Then, Pearson’s (for normal distribution) or Spearman’s (for non-normal distribution) correlation analysis was applied to explore relationships among HDRS-17 scores, age, education level, age of tremor onset, tremor duration, and scores on TRS parts A & B, TRS part C, TETRAS, TETRAS-ADL, MMSE and HARS-14 in the depressed ET patients.

In consideration of potential confounding factors, the age, sex, years of education, gray matter volume, and MMSE and HARS-14 scores of each subject were used as covariates in all of the above analyses, and the TRS parts A & B and TRS part C scores were also considered as covariates for analyses between the depressed ET and non-depressed ET patients.

## Results

### Demographic and clinical characteristics

Demographic and clinical information is shown in Table 1, and the age, education level, tremor of onset, and scores on TRS parts A & B, TRS part C, TETRAS, TETRAS-ADL, MMSE, HDRS-17 and HARS-14 in depressed ET patients showed a normal distribution (*P* = 0.63, 0.12, 0.32, 0.66, 0.33, 0.45, 0.27, 0.06, 0.97 and 0.06, respectively), and the tremor duration in the depressed ET patients showed a non-normal distribution (*P* = 0.035). Among these clinical data, a significant correlation was observed between TRS parts A & B and TETRAS and between TRS part C and TETRAS-ADL (Pearson’s: *r* = 0.61 *P* = 3.542E-5; *r* = 0.47 *P* = 0.003), and a marginally significant correlation was observed between HDRS-17 scores and education level (Pearson’s *r* = 0.27 *P* = 0.09) in the depressed ET patients.

**Table 1:** Demographic and clinical features of depressed ET, non-depressed ET and HCs

Measure	DET (41)	ET (49)	HCs (43)	Statistic	*P value*		
					DET v HCs	ET v HCs	DET v ET
Demographic							
Age	47.85 ± 15.66	47.08 ± 12.657	46.91 ± 14.67	F = 0.24	0.56	0.95	0.55
Sex (male/female)	26:15	32:17	27:16	x^2^ = 0.07	0.95	0.80	0.85
Education (year)	14.73 ± 4.17	11.57 ± 4.12	12.39 ± 3.97	F = 6.98	3.82E-4	3.82E-4	0.01
Handedness (R/L)	41:0	49:0	43:0				
Clinical of psychology and cognitive							
HDRS-17	18.98 ± 6.35	3.53 ± 1.46	1.93 ± 1.14	F = 275.25	4.03E-29	9.7E-8	1.01E-28
MMSE	26.00 ± 1.40	26.46 ± 1.52	29.02 ± 1.10	F = 61.91	6.71E-18	1.83E-14	0.13
HARS-14	7.56 ± 3.47	4.02 ± 1.57	1.49 ± 0.91	F = 79.71	5.67E-18	9.36E-15	7.31E-9
Clinical of tremor							
Tremor of onset (year)	34.93 ± 11.84	34.43 ± 10.16	NA	T = 0.21	NA	NA	0.83
Tremor duration (year)	13.90 ± 9.40	12.69 ± 7.02	NA	T = 0.70	NA	NA	0.49
TRS-parts A&B	22.66 ± 7.28	20.90 ± 7.35	NA	T = 1.14	NA	NA	0.26
TRS-part C	14.05 ± 4.74	11.82 ± 5.80	NA	T = 1.97	NA	NA	0.05
TETRAS	18.32 ± 8.27	17.63 ± 6.75	NA	T = 0.57	NA	NA	0.57
TETRAS-ADL	21.39 ± 6.51	20.91 ± 5.66	NA	T = 1.31	NA	NA	0.19

*DET* depressed ET, *ET* essential tremor, *HCs* healthy controls, *HDRS-17* 17-item Hamilton Depression Rating Scale, *MMSE* Mini-Mental State Examination, *HARS-14* 14-item Hamilton Anxiety Rating Scale, *TRS* Fahn-Tolosa-Marin Tremor Rating Scale, *TETRAS* Essential Tremor Rating Assessment Scale, *TETRAS-ADL* Essential Tremor Rating Assessment Scale-Activities of Daily Living

### Local FC changes among the three groups

**Fig. 1:**
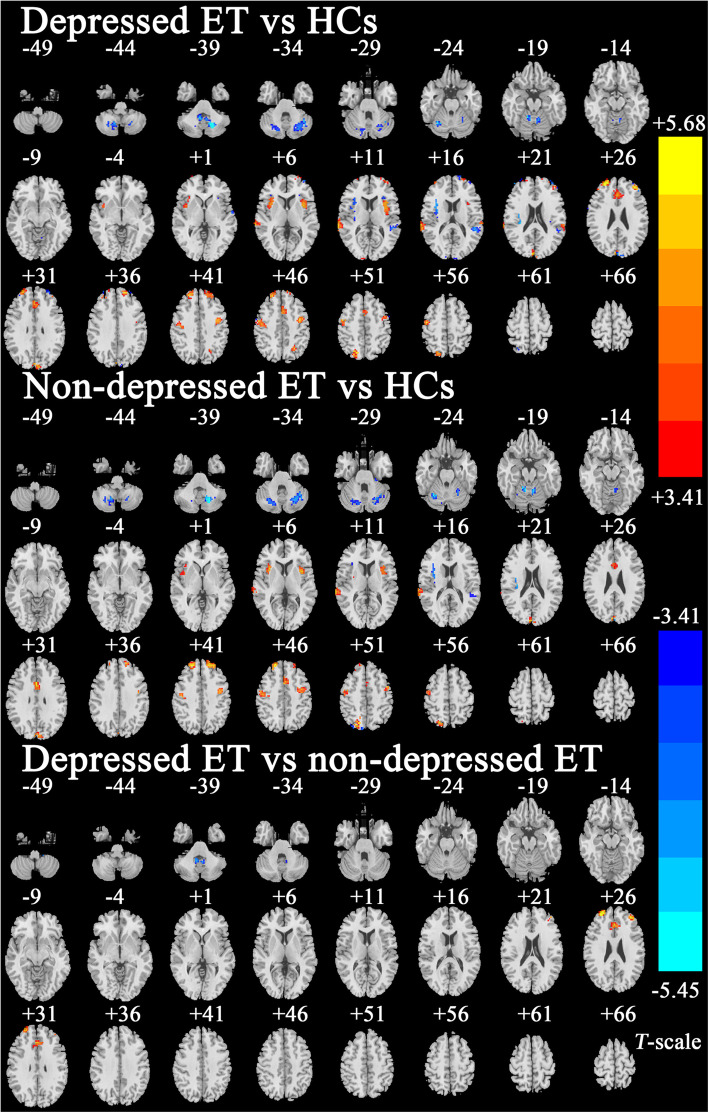
Post-hoc two-sample *t*-test results of ReHo changes among depressed ET, non-depressed ET and HCs. GRF corrected with a voxel-level *P* < 0.001 and a cluster-level *P* < 0.05, ANOVA results as mask, estimated smoothing kernel with FWHM: 6 × 6 × 4 mm3 and cluster size > 432 mm3. ReHo: regional homogeneity, ET: essential tremor, HCs: healthy controls

One-way ANOVA results (GRF corrected with a voxel-level *P* < 0.01 and a cluster-level *P* < 0.05, gray matter mask, estimated smoothing kernel with FWHM: 6 × 6 × 4 mm3 and cluster size > 405 mm^3^) showed ReHo changes in the prefrontal cortices, anterior cingulate cortices, motor cortices, insular cortices, superior parietal lobules, superior temporal cortices, cuneus and cerebellum among the three groups (Fig. S1). Figure 1 shows the post hoc two-sample *t*-test results (GRF corrected with a voxel-level *P* < 0.001 and a cluster-level *P* < 0.05, ANOVA results as mask, estimated smoothing kernel with FWHM: 6 × 6 × 4 mm^3^ and cluster size > 432 mm^3^). Compared with the HCs, the depressed ET patients had increased ReHo in the bilateral precentral cortices, supplementary motor cortices, superior and middle prefrontal cortices, anterior cingulate cortices, insular cortices, superior parietal lobules, cuneus and right superior temporal cortex, and decreased ReHo in the bilateral cerebellum lobules IV-V, cerebellum lobules VI, cerebellum lobules VIII, cerebellum lobules IX, cerebellum lobules crus 1 and left superior temporal cortex. Compared with the HCs, the non-depressed ET patients showed similar ReHo differences to the depressed ET patients, but increased ReHo in the bilateral middle prefrontal cortices and anterior cingulate cortices and decreased ReHo in the bilateral cerebellum lobules IX were not obvious. Compared with the non-depressed ET patients, the depressed ET patients showed increased ReHo in the middle prefrontal cortices and anterior cingulate cortices and decreased ReHo in the bilateral cerebellum lobules IX. The peak Montreal Neurological Institute coordinates [17] are listed in Table S1.

### Inter-ROI FC changes among the three groups

**Fig. 2:**
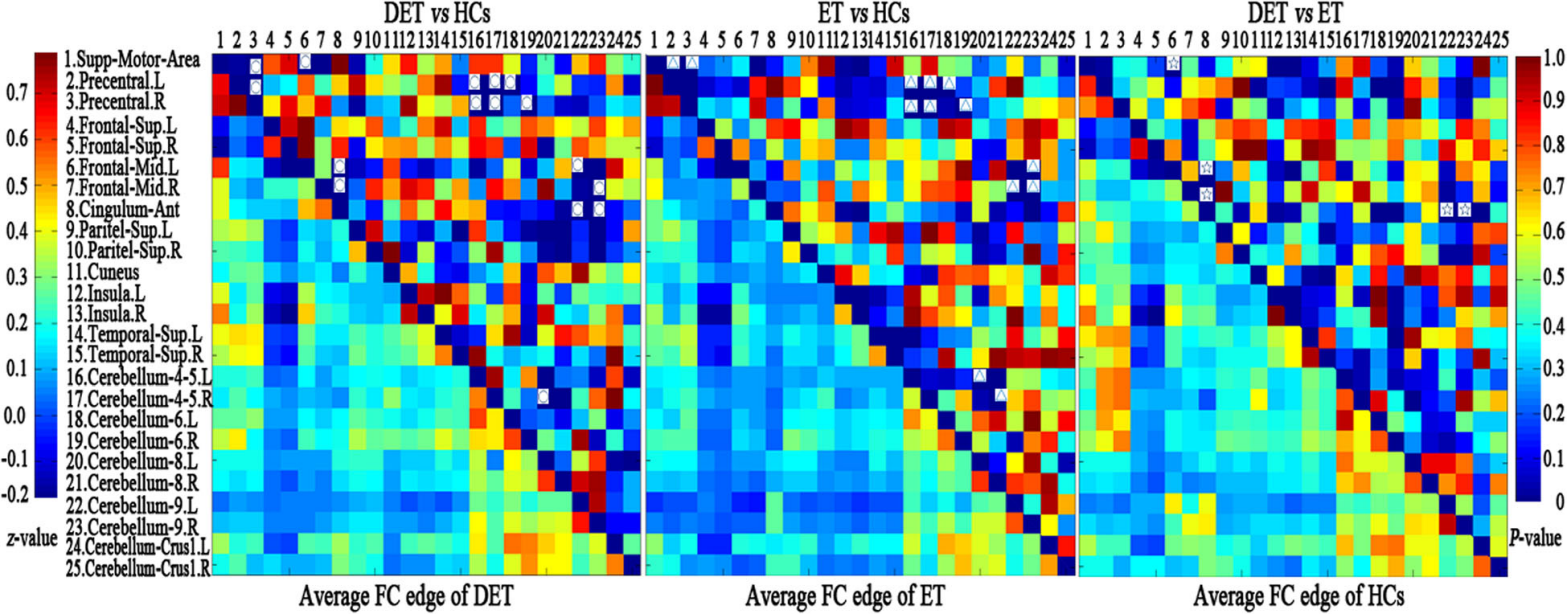
Post-hoc two-sample *t*-test results of matrix FC changes among depressed ET, non-depressed ET and HCs. Bonferroni multiple comparison corrections, corrected *P* < 0.05/17*9. **Right upper**: the *P*-value matrix of DET vs HCs, ET vs HCs, and DET vs ET. **Left lower**: the average FC matrix of DET, ET and HCs. ○ indicated the significant changes inter-ROI FC in DET vs HCs, △ indicated the significant changes inter-ROI FC in ET vs HCs, ☆ indicated the significant changes inter-ROI FC in DET vs ET. ROI: regions of interest, FC: functional connectivity, DET: depressed essential tremor, ET: essential tremor, HCs: healthy controls

Twenty-five significant changes in ReHo clusters were observed in the depressed ET patients compared with the HCs, and these 25 ROIs were defined (the peak MNI coordinates are listed in Table S2). One-way ANOVA results (Bonferroni multiple comparison corrections, corrected *P* < 0.05/25*12) showed 18 paired ROIs with significant changes in inter-ROI FC. Figure 2 shows the post hoc two-sample *t*-test results (Bonferroni’s multiple comparison corrections, corrected *P* < 0.05/17*9). Compared with the HCs, the depressed ET patients showed increased inter-ROI FC between the supplementary motor cortex and right precentral cortex, between the right precentral cortex and left precentral cortex, between the supplementary motor cortex and left middle prefrontal cortex, and between the anterior cingulate cortex and bilateral middle prefrontal cortices and decreased inter-ROI FC between the bilateral cerebellum lobules IV-V and bilateral precentral cortex, the bilateral cerebellum lobules VI and bilateral precentral cortex, the bilateral middle prefrontal cortices and bilateral cerebellum lobules IX, between the anterior cingulate cortex and bilateral cerebellum lobules IX and between the right cerebellum lobules IV-V and left cerebellum lobules VIII. Compared with the HCs, the non-depressed ET patients showed similar differences to the depressed ET patients, but increased inter-ROI FC between the supplementary motor cortex and left middle prefrontal cortex and between the anterior cingulate cortex and bilateral middle prefrontal cortices, while decreased inter-ROI FC between the anterior cingulate cortex and bilateral cerebellum lobules IX were not obvious. Compared with the non-depressed ET patients, the depressed ET patients showed increased inter-ROI FC between the supplementary motor cortex and left middle prefrontal cortices and between the anterior cingulate cortex and bilateral middle prefrontal cortices and decreased inter-ROI FC between the anterior cingulate cortex and bilateral cerebellum lobules IX.

### FC-depression correlations

**Fig. 3:**
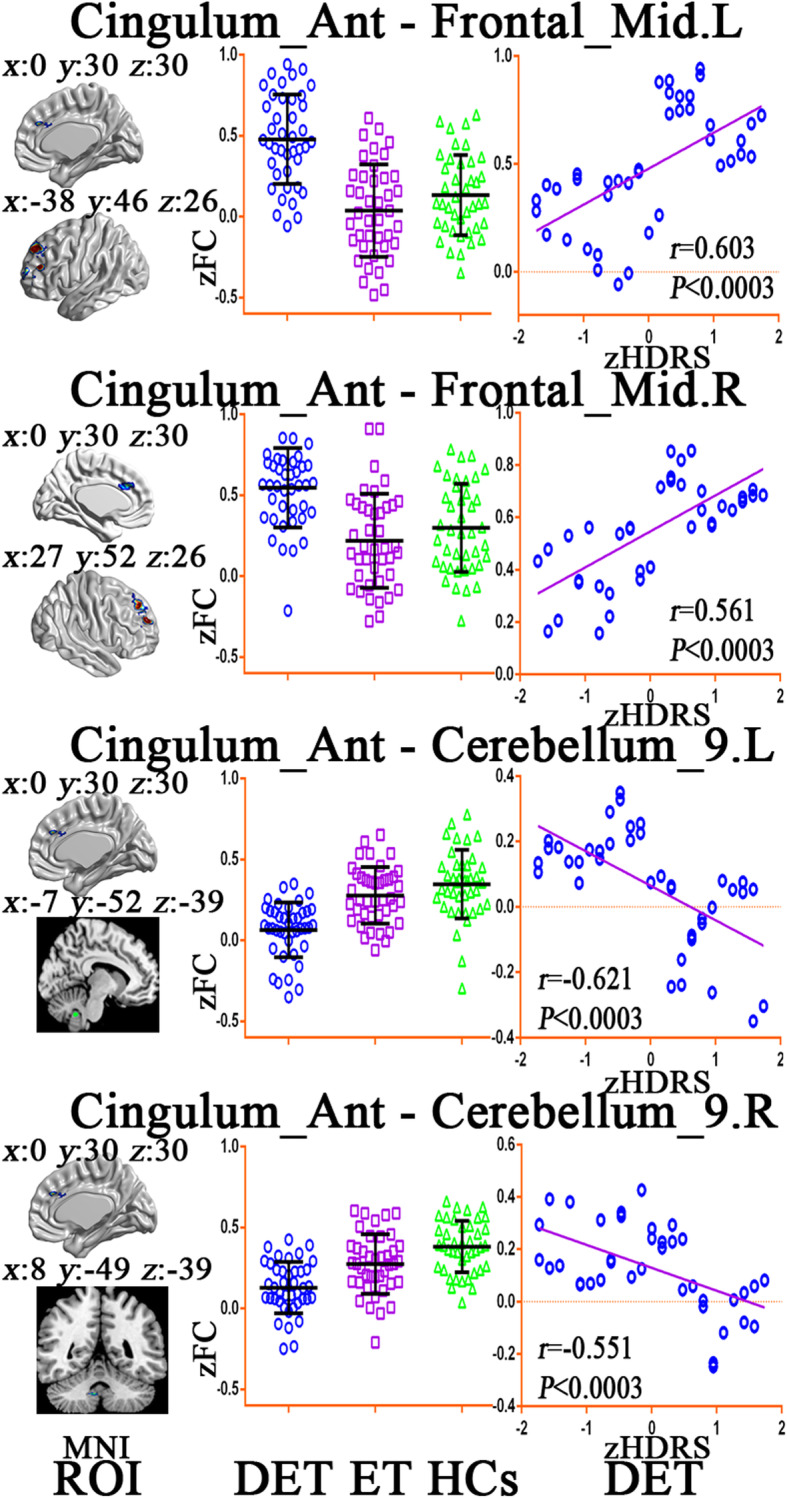
Correlation analysis results between depression severity and significant inter-ROI FC changes in depressed ET patients. Bonferroni multiple comparison corrections, corrected *P <* 0.05/15*8. **Left**: the inter-ROI MNI location, **middle**: the inter-ROI FC values among DET, ET and HCs groups, **right**: the scatter plots for the correlation analysis in the depressed ET patients. Cingulum Ant: anterior cingulate cortex, Frontal Mid: middle prefrontal gyrus, L: left, R: right, ROI: regions of interest, zFC: z transforms functional connectivity, zHDRS: z transforms Hamilton Depression Rating Scale scores, DET: depressed essential tremor, ET: essential tremor, HCs: healthy controls

The voxel-wise Pearson’s correlation analysis showed that no brain area ReHo values were associated with depression severity in depressed ET patients. However, the ROI-wise Pearson’s correlation analyses showed a positive correlation of inter-ROI FC values of the anterior cingulate cortex and bilateral middle prefrontal cortices with the HDRS-17 scores in the depressed ET patients and negative correlation of inter-ROI FC values of anterior cingulate cortex and bilateral cerebellum lobules IX with the HDRS-17 scores of the depressed ET (Fig. 3) (Bonferroni’s multiple comparison corrections, corrected *P* < 0.05/15*8).

## Discussion

To the best of our knowledge, this study is the first to investigate local and inter-ROI FC changes in depressed ET patients, and three main findings were obtained: 1) compared with the HCs and non-depressed ET patients, the depressed ET patients showed local FC changes mostly in the bilateral middle prefrontal cortices, anterior cingulate cortices and cerebellum lobules IX; 2) inter-ROI FC changes were also observed in the frontal-cerebellar-anterior cingulate cortex pathway; and 3) inter-ROI FC changes between the cerebellum lobules IX and anterior cingulate cortices and between the middle prefrontal cortices and anterior cingulate cortices were correlated with clinical depression severity in the depressed ET patients. The following discussion focuses on the main findings regarding the frontal-cerebellar-anterior cingulate cortex circuits in depressed ET patients.

### Depression can be a primary phenomenon in ET

It is still debated whether depression is an intrinsic part of ET or a secondary phenomenon. Chandran et al. [18] reported that HDRS scores were correlated with tremor severity scores in depressed ET patients. Wharen et al. [19] observed that deep brain stimulation of the ventralis intermedius nucleus of the thalamus significantly improved tremor and depression in patients with severe ET. These studies seem to support that depression in ET is merely a secondary phenomenon in response to tremor severity. However, many studies [4, 20] have not observed this correlated relationship between tremor and depression. Consistent with these studies, our study failed to find any significant correlation between depression severity and other clinical characteristics, including tremor severity, in depressed ET patients. More importantly, our findings showed that the local and inter-ROI FC changes were not confined to the same cerebellar-cortical pathway as those in the non-depressed ET patients, but they extended to various other brain regions, including the middle prefrontal cortices, anterior cingulate cortices and cerebellum lobules IX. These FC changes involving other brain areas in the depressed ET patients were similar to those observed in patients with neurodegenerative diseases involving overlapping symptoms, namely PD with depression, and all studies [21] agree that depression in PD is a consequence of the neurodegenerative process. Therefore, we suggest that depression in ET might be a primary manifestation of the disease rather than secondary to impaired motor functions and decreased quality of life due to tremor.

### The frontal-cerebellar-anterior cingulate cortex circuit can be a key pathogenic pathway associated with depression in ET patients

Emotional regulation and cognitive processing are a complex system of information exchange, and these modulatory activities have been associated with various brain areas, especially those in the limbic-cortical-striatal-pallidal-thalamic circuits [22]. Dysfunction in these brain areas has been linked to depressive symptoms, and traditionally, studies [23] have paid close attention to the frontal-limbic network. The most recent evolution of the frontal-striatal-cerebellar circuit model has gained increasing attention, and this model emphasizes that the posterior cerebellum lobe, anterior cingulate cortex and ventromedial prefrontal cortex, as vital components of this circuit might be associated with depression-related functional impairments, such as problem solving, decision making, self-referential problems and negative ruminations [22, 24]. Studies [24–26] using electroencephalography (EEG), magnetoencephalography (MEG), positron emission tomography (PET), single-photon emission computed tomography (SPECT), magnetic resonance spectroscopy (MRS), structural and functional magnetic resonance imaging have demonstrated that abnormalities in frontal-striatal-cerebellar circuits are associated with major depressive disorder, ET with depression, PD with depression and other chronic diseases with depression, such as chronic pain with depression. Consistent with these studies, our findings also showed that not only local FC but also inter-ROI FC changes in the frontal-cerebellar-anterior cingulate cortex circuit are involved in depressed ET patients. It seems difficult for us to understand that these FC change patterns are linked to depression in ET patients, and the greatest difficulty is that the pathogenesis of ET remains unclear. However, recently converging evidence from post-mortem [27] and PET [28] studies has indicated that disruption of the gamma-aminobutyric acid (GABA) system, especially involving the cerebellum, thalamus and cortices, plays a pathogenic role, while previous studies [29] have demonstrated that dysfunctions in the GABAergic system have a close association with depression. Therefore, we suggest that the frontal-cerebellar-anterior cingulate cortex circuit FC changes might be the result of GABAergic dysfunction, and this circuit functions as a key pathogenic pathway associated with depression in ET patients.

### Limitations

There are some limitations of the present study. First, we demonstrated that not only local FC but also inter-ROI FC changes in the frontal-cerebellar-anterior cingulate cortices circuit were associated with depressed ET patients. However, some other data-driven analysis methods of RS-fMRI, such as those based on a graph theoretical approach with a whole-brain FC analysis, might provide more useful information about FC changes in depressed ET patients. Despite this fact, we focused only on local FC changes, which are among the most common data-driven analysis methods of RS-fMRI, since this method could more directly reveal the FC changes in depressed ET patients. Second, although based on our findings, we strongly suggested that depression could be a primary phenomenon in ET, this study was cross-sectional, and follow-up data might be better able to directly address these issues. Third, all of the depressed and non-depressed ET patients were recruited from the outpatient department, and our findings might not capture all of the aspects of the depressive characteristics in ET patients. In the future, a population-based study will be performed. Finally, due to the absence of biological and pathogenic markers, the diagnosis of ET relied only on clinical phenomenology and neurologic examinations, and misdiagnosis is common. However, all of the ET patients had long follow-up periods to minimize the risk of misdiagnosis in our study.

## Conclusion

Using local and inter-ROI FC analysis, we emphasized that the local and inter-ROI FC changes in the frontal-cerebellar-anterior cingulate cortices circuit were involved in depressed ET patients, and in this circuit, the bilateral middle prefrontal cortices, anterior cingulate cortices and cerebellum IX might function as crucial pathogenic structures.

## Supplementary Information

**Additional file 1 Fig. S1**. ANOVA results of ReHo analyses changes among depressed ET, non-depressed ET and HCs. GRF corrected with a voxel-level *P* < 0.01 and a cluster-level *P* < 0.05, grey matter mask, estimated smoothing kernel with FWHM: 6 × 6 × 4 mm3 and cluster size > 405 mm3. ReHo: regional homogeneity, ET: essential tremor, HCs: healthy controls. **Table S1**. The brain regions and peak MNI coordinates of significant difference clusters in ReHo analysis among depressed ET, non-depressed ET and HCs. MNI: Montreal Neurological Institute, ReHo: regional homogeneity, ET: essential tremor, HCs: healthy controls. **Table S2**. The brain regions and peak MNI coordinates of significant changes ReHo clusters. MNI: Montreal Neurological Institute, ReHo: regional homogeneity.

## Data Availability

The datasets used and analysed during the current study are available from the corresponding author on reasonable request.

## Abbreviations

ET: Essential tremor
HCs: Healthy controls
ReHo: Regional homogeneity
FC: Functional connectivity
ROI: Regions of interest
MNI: Montreal Neurological Institute
