# Supplementary material for: Altered local and matrix functional connectivity in depressed essential tremor patients
Source: BMC Neurol. 2021 Feb 11;21:68. doi: 10.1186/s12883-021-02100-3 (PMC7879612; doi:10.1186/s12883-021-02100-3)
Supplement: Supplementary file 1 — Additional file 1 Fig. S1. ANOVA results of ReHo analyses changes among depressed ET, non-depressed ET and HCs. GRF corrected with a voxel-level P < 0.01 and a cluster-level P < 0.05, grey matter mask, estimated smoothing kernel with FWHM: 6 × 6 × 4 mm3 and cluster size > 405 mm3. ReHo: regional homogeneity, ET: essential tremor, HCs: healthy controls. Table S1. The brain regions and peak MNI coordinates of significant difference clusters in ReHo analysis among depressed ET, non-depressed ET and HCs. MNI: Montreal Neurological Institute, ReHo: regional homogeneity, ET: essential tremor, HCs: healthy controls. Table S2. The brain regions and peak MNI coordinates of significant changes ReHo clusters. MNI: Montreal Neurological Institute, ReHo: regional homogeneity. [file 12883_2021_2100_MOESM1_ESM.docx]

**Altered local and matrix functional connectivity in depressed essential tremor patients**

Xiyue Duan^aŢ^, Zhou Fang^aŢ^, Li Tao^a^, Huiyue Chen^a^, Xiaoyu Zhang^a^, Yufen Li^a^, Hansheng Wang^a^, Aotian Li^a^, Xueyan, Zhang^a^, Ya Pang^a^, Min Gu^a^, Jiahui Wu^a^, Fajin Lv, ^a^, Tianyou Luo^a^, Oumei Cheng^b^, Jin Luo^b^, Zheng Xiao^b^, Weidong Fang^a^*

^a^Department of Radiology, The First Affiliated Hospital of Chongqing Medical University, Chongqing 400016, China

^b^Department of Neurology, The First Affiliated Hospital of Chongqing Medical University, Chongqing 400016, China

^Ţ^Co-first author: These authors contributed equally to the manuscript

**Text S1 Image Acquisition**

All MR images were acquired using a GE Signa Hdxt 3-T scanner (General Electric Medical Systems, Waukesha, WI) equipped with a standard 8-channel head coil. Foam padding and earplugs were used to minimize head motion and to reduce scanner noise. During RS-fMRI scanning, all subjects were told to relax, to remain still with their eyes closed, and to remain awake (which was immediately confirmed via post-scan debriefing). RS-fMRI data were acquired using an echo-planar imaging (EPI) pulse sequence with the following parameters: 33 axial slices, slice thickness/gap = 4.0/0 mm, matrix = 64 × 64, TR = 2000 ms, TE = 40 ms, flip angle = 90º, FOV = 240 × 240 mm, and a total of 240 volumes were obtained (duration = 8 minutes). High-resolution 3D T1-weighted images (TR = 8.3 ms, TE = 3.3 ms, flip angle = 15°, slice thickness/gap = 1.0/0 mm, FOV = 240 × 240 mm, and matrix = 256 × 192). DTI (TR = 15000 ms, TE = 86.2 ms, thickness/gap = 2.4/0 mm, FOV = 240 × 240 mm, and matrix = 130 × 128, b = 1000 s/mm2, 38 gradient directions including 8 b = 0 gradient directions) and T2-weighted FLAIR images (TR = 8000 ms, TE = 126 ms, TI = 1500 ms, slice thickness/gap = 5.0/1.5 mm, FOV = 240 × 240 mm, and matrix = 256 × 192) were also acquired. We did not use the T2-weighted FLAIR images for data processing, but they were used for image evaluation and data quality assessment (see Quality assurance).

**Text S2 The detailed data preprocessing steps**

The data preprocessing consisted of the following steps:

1. *Removal of the first 10 time points*: For scanner stabilization and the subjects’ acclimating to the MR scanning environment, the first 10 volumes were discarded, and the remaining 230 time points included in the subsequent data preprocessing.

2. *Slice timing correction:* This was used to correct for different acquisition time across slices in a volume.

3. *Realignment:* This was used to realign the subsequent functional images to the first volume to correct for within-run head motions, and resulting in six rigid-body head motion parameters. These parameters were employed to assess head movement and ensure the quality of RS-fMRI data.

4. *T1 segmentation and spatial normalization:* 3D T1-weighted images were segmented into grey matter (GM), white matter (WM), and cerebrospinal fluid (CSF) probability maps using SPM dartel segmentation. All the GM, WM and CSF images were resampled to 1.5 × 1.5 × 1.5 mm^3^ and, spatially normalized to the MNI space using both affine transformation and non-linear deformation, after that, resampled to 3 × 3 × 3 mm^3^ voxel resolution with RS-fMRI and the deformation field was applied to the RS-fMRI data (before step 4, the 3D-T1 images were co-registered to the mean RS-fMRI data for each subject).

5. Regression out six head motion parameters and the mean time series of global, white matter and cerebrospinal fluid signals.

6. *Smoothing:* Spatial smoothing with a Gaussian kernel of 4 mm full width at half maximum

7. *Detrending and filtering:* These steps removed the extremely low frequency drift and the high-frequency physiological noises. For detrending we used 1^st^ order polynomial functions; and for filtering, we adopted band-pass filtering (0.01 Hz < *f* < 0.08 Hz) to the time series for each voxel.

Note: for ReHo calculation without perform step 6 in data preprocessing steps and for inter-ROIs FC analysis perform all the above steps in data preprocessing steps.

**Text S3 Head motion and imaging quality control**

we adopt a strict criterion for head motion, a mean framewise displacement (FD) < 0.05 mm and a frame-wise displacement of head movement < 50%. The imaging quality control (QC) performed in preprocessing show that all of the imaging scores are 5, indicating very good imaging QC.

**Figure S1. ANOVA results of ReHo analyses changes among depressed ET, non-depressed ET and HCs.**


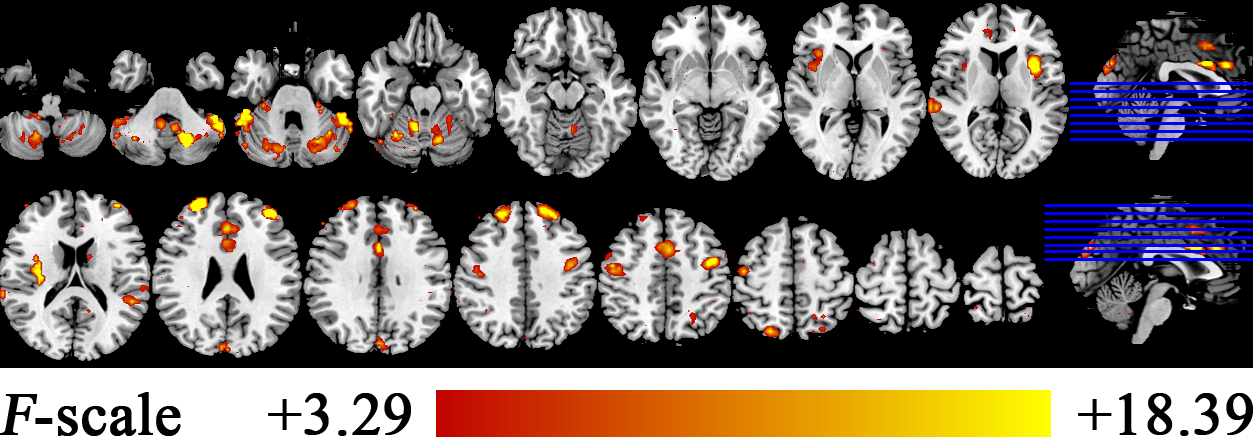


GRF corrected with a voxel-level *P* < 0.01 and a cluster-level *P* < 0.05, grey matter mask, estimated smoothing kernel with FWHM: 6 × 6 × 4 mm3 and cluster size > 405 mm3. ReHo: regional homogeneity, ET: essential tremor, HCs: healthy controls.

**Table S1. The brain regions and peak MNI coordinates of significant difference clusters in ReHo analysis among depressed ET, non-depressed ET and HCs.**

| Brain region | Size (voxels) | *T*-value | MNI coordinates | | |
| --- | --- | --- | --- | --- | --- |
|  |  |  | *x* | *y* | *z* |
| **Depressed ET versus HCs** | | | | | |
| Left supplementary motor cortex | 57 | 4.69 | 0 | 9 | 45 |
| Right supplementary motor cortex | 39 | 4.18 | 2 | 10 | 47 |
| Left precentral cortex | 61 | 5.21 | -43 | -3 | 48 |
| Right precentral cortex | 73 | 5.13 | 48 | -7 | 46 |
| Left superior prefrontal gyrus | 49 | 4.96 | -21 | 46 | 43 |
| Right superior prefrontal gyrus | 53 | 5.14 | 18 | 46 | 43 |
| Left middle prefrontal gyrus | 43 | 5.35 | -38 | 46 | 26 |
| Right middle prefrontal gyrus | 54 | 5.68 | 27 | 52 | 26 |
| Left anterior cingulum cortex | 59 | 4.29 | 0 | 30 | 30 |
| Right anterior cingulum cortex | 48 | 3.75 | 2 | 33 | 26 |
| Left superior parietal lobule | 34 | 3.71 | -27 | -60 | 45 |
| Right superior parietal lobule | 41 | 3.52 | 18 | -69 | 51 |
| Left cuneus | 21 | 3.67 | -5 | -88 | 29 |
| Right cuneus | 16 | 3.49 | 7 | -87 | 26 |
| Left insular | 79 | 4.53 | -33 | 9 | 9 |
| Right insular | 63 | 4.96 | 36 | 12 | 6 |
| Left superior temporal gyrus | 45 | -3.61 | -50 | -43 | 18 |
| Right superior temporal gyrus | 52 | 4.27 | 67 | -34 | 13 |
| Left cerebellum IV~V | 36 | -4.75 | -9 | -53 | -18 |
| Right cerebellum IV~V | 39 | -4.63 | 15 | -48 | -18 |
| Left cerebellum VI | 35 | -4.69 | -24 | -55 | -27 |
| Right cerebellum VI | 47 | -4.82 | 28 | -63 | -27 |
| Left cerebellum VIII | 78 | -4.27 | -18 | -66 | -39 |
| Right cerebellum VIII | 70 | -4.79 | 18 | -66 | -42 |
| Left cerebellum IX | 49 | -5.45 | -7 | -52 | -39 |
| Right cerebellum IX | 29 | -5.06 | 8 | -49 | -39 |
| Left cerebellum crus 1 | 53 | -4.78 | -32 | -64 | -33 |
| Right cerebellum crus 1 | 47 | -4.32 | 23 | -71 | -33 |
| **Non depressed ET versus HCs** | | | | | |
| Left supplementary motor cortex | 51 | 4.26 | -2 | 11 | 45 |
| Right supplementary motor cortex | 27 | 4.06 | 3 | 11 | 49 |
| Left precentral cortex | 49 | 4.44 | -47 | -2 | 51 |
| Right precentral cortex | 63 | 4.88 | 45 | -12 | 54 |
| Left superior prefrontal gyrus | 61 | 5.45 | -24 | 45 | 39 |
| Right superior prefrontal gyrus | 53 | 5.36 | 21 | 45 | 39 |
| Left anterior cingulum cortex | 21 | 3.51 | 0 | 12 | 30 |
| Right anterior cingulum cortex | 17 | 3.43 | 5 | 16 | 28 |
| Left superior parietal lobule | 20 | 3.81 | -24 | -60 | 46 |
| Right superior parietal lobule | 37 | 3.52 | 19 | -73 | 50 |
| Left cuneus | 27 | 5.35 | 3 | -87 | 24 |
| Right cuneus | 19 | 4.21 | 5 | -90 | 22 |
| Left insular | 67 | 5.22 | -33 | 9 | 9 |
| Right insular | 53 | 4.71 | 33 | 15 | 6 |
| Left superior temporal gyrus | 45 | -3.61 | -50 | -43 | 18 |
| Right superior temporal gyrus | 49 | 3.47 | 66 | -29 | 10 |
| Left cerebellum IV~V | 50 | -4.74 | -8 | -54 | -18 |
| Right cerebellum IV~V | 39 | -5.40 | 9 | -51 | -18 |
| Left cerebellum VI | 53 | -4.95 | -23 | -53 | -26 |
| Right cerebellum VI | 49 | -3.87 | 25 | -62 | -26 |
| Left cerebellum VIII | 81 | -5.21 | -18 | -62 | -43 |
| Right cerebellum VIII | 70 | -5.19 | 15 | -65 | -43 |
| Left cerebellum crus 1 | 37 | -5.01 | -40 | -55 | -33 |
| Right cerebellum crus 1 | 51 | -3.98 | 26 | -69 | -33 |
| **Depressed ET versus non depressed ET** | | | | | |
| Left middle prefrontal gyrus | 37 | 5.43 | -39 | 45 | 27 |
| Right middle prefrontal gyrus | 43 | 5.61 | 30 | 54 | 27 |
| Left anterior cingulum cortex | 27 | 4.82 | 0 | 33 | 27 |
| Right anterior cingulum cortex | 29 | 3.90 | 3 | 27 | 29 |
| Left cerebellum IX | 33 | -4.49 | -9 | -51 | -36 |
| Right cerebellum IX | 31 | -4.33 | 6 | -49 | -40 |

MNI: Montreal Neurological Institute, ReHo: regional homogeneity, ET: essential tremor, HCs: healthy controls.

**Table S2. The brain regions and peak MNI coordinates of significant changes ReHo clusters.**

| Brain region | MNI coordinates | | |
| --- | --- | --- | --- |
|  | *x* | *y* | *z* |
| Supp-Motor-Area (Supplementary motor cortex) | 0 | 9 | 45 |
| Precentral.L (Left precentral cortex) | -43 | -3 | 48 |
| Precentral.R (Right precentral cortex) | 48 | -7 | 46 |
| Frontal-Sup.L (Left superior prefrontal gyrus) | -21 | 46 | 43 |
| Frontal-Sup.R (Right superior prefrontal gyrus) | 18 | 46 | 43 |
| Frontal-Mid.L (Left middle prefrontal gyrus) | -38 | 46 | 26 |
| Frontal-Mid.R (Right middle prefrontal gyrus) | 27 | 52 | 26 |
| Cingulum-Ant (Anterior cingulum cortex) | 0 | 30 | 30 |
| Paritel-Sup.L (Left superior parietal lobule) | -27 | -60 | 45 |
| Paritel-Sup.R (Right superior parietal lobule) | 18 | -69 | 51 |
| Cuneus | -5 | -88 | 29 |
| Insula.L (Left insular) | -33 | 9 | 9 |
| Insula.R (Right insular) | 36 | 12 | 6 |
| Temporal-Sup.L (Left superior temporal gyrus) | -50 | -43 | 18 |
| Temporal-Sup.R (Right superior temporal gyrus) | 67 | -34 | 13 |
| Cerebellum-4-5.L (Left cerebellum IV~V) | -9 | -53 | -18 |
| Cerebellum-4-5.R (Right cerebellum IV~V) | 15 | -48 | -18 |
| Cerebellum-6.L (Left cerebellum VI) | -24 | -55 | -27 |
| Cerebellum-6.R (Right cerebellum VI) | 28 | -63 | -27 |
| Cerebellum-8.L (Left cerebellum VIII) | -18 | -66 | -39 |
| Cerebellum-8.R (Right cerebellum VIII) | 18 | -66 | -42 |
| Cerebellum-9.L (Left cerebellum IX) | -7 | -52 | -39 |
| Cerebellum-9.R (Right cerebellum IX) | 8 | -49 | -39 |
| Cerebellum-Crus.L (Left cerebellum crus 1) | -32 | -64 | -33 |
| Cerebellum-Crus.R (Right cerebellum crus 1) | 23 | -71 | -33 |

MNI: Montreal Neurological Institute, ReHo: regional homogeneity.
